# Characteristics of Patients Returning to Work After Brain Tumor Surgery

**DOI:** 10.3389/fnhum.2020.609080

**Published:** 2021-02-03

**Authors:** Silvia Schiavolin, Arianna Mariniello, Morgan Broggi, Francesco Acerbi, Marco Schiariti, Angelo Franzini, Francesco Di Meco, Paolo Ferroli, Matilde Leonardi

**Affiliations:** ^1^Neurology, Public Health and Disability Unit, Fondazione IRCSS Istituto Neurologico Carlo Besta, Milan, Italy; ^2^Department of Neurosurgery, Fondazione IRCSS Istituto Neurologico Carlo Besta, Milan, Italy

**Keywords:** return to work, neurosurgery, meningioma, glioma, patient reported outcome measures, cognitive evaluation

## Abstract

**Objective:** To investigate the differences between patients returning to work and those who did not after brain tumor surgery.

**Methods:** Patients were evaluated before surgery and after 3 months. The Montreal Cognitive Assessment test, Trail-Making Test (parts A and B), 15-word Rey–Osterrieth Word List (immediate and delayed recall), F-A-S tests, and Karnosfky Performance Status were used to assess cognitive status, attention, executive functions, memory, word fluency, and functional status. Patient-reported outcome measures (PROMs) used to evaluate emotional distress and disability were the Hospital Anxiety and Depression Scale and World Health Organization Disability Assessment Schedule. Clinical and work-related variables, PROMs, and cognitive tests were compared using chi-squared, *t*-test or Mann–Whitney *U* test.

**Results:** Sixty patients were included. Patients returning to work were 61.3 and 31.0% among people with meningioma and glioma, respectively. They reported lower postoperative disability and lesser home-to-work travel time. Patients with meningioma also showed better preoperative and postoperative attention and executive functions, better postoperative functional and cognitive status, and lower frequency of treatments.

**Conclusions:** These variables should be considered in a clinical context to plan interventions for people who need support during return to work and in future research to investigate preoperative and postoperative predictive factors of going back to work.

## Introduction

The number of cancer survivors continues to grow as a consequence of different factors, e.g., the improvement of treatments, earlier diagnosis, and a better access to health care (Ries et al., 2007; Siegel et al., 2019). Living with cancer often means dealing with physical, cognitive, or psychological difficulties, and consequently, return to work may be a challenge for these patients. Previous studies report brain and central nervous system cancers among tumors that are more associated with job loss, reduction in earnings, and greater levels of work limitations (Mehnert, 2011). Among the adult brain tumors, meningiomas and malignant gliomas are the most common, accounting for 37.6 and 25.5% of all tumors, respectively (Ostrom et al., 2019). The majority of cerebral meningiomas are benign without a significant risk of death although skull-based meningiomas can cause severe morbidity and those atypical and malignant can be associated with mortality, high rate of recurrence, and significant deficits. Most meningioma patients are faced with cognitive deficits prior to surgery and tend to improve following surgery even if an impairment in a wide range of cognitive functions can remain compared with healthy controls (Meskal et al., 2016). Gliomas range from benign grade I tumors to locally aggressive grade IV and usually require adjuvant postoperative treatments as opposed to meningioma. The most prevalent symptoms influencing quality of life and working abilities are seizures, cognitive deficits, drowsiness, dysphagia, headache, confusion, aphasia, motor deficits, fatigue, and dyspnea (Ijzerman-Korevaar et al., 2018). Among cognitive functions, memory and executive functions seem to be the most frequently affected before surgery in patients with glioma, and a low incidence of additional deficits and early improvement can be observed after surgery. Furthermore, language is also frequently disturbed in glioma patients (Talacchi et al., 2011).

The majority of previous studies about employment status focused on job loss and working difficulties of cancer survivors of breast or mixed cancer populations, reporting an overall rate of return to work of 63.5% (Mehnert, 2011). Few studies on brain tumors were conducted, mainly focusing on gliomas and reporting the employment rates after surgical treatment that range from 44 to 80% (Rusbridge et al., 2013). Some of these studies analyze factors associated with return to work, highlighting the positive effect of younger age, fewer comorbidities, higher occupation categories, better functional status, sole breadwinner status, lower tumor volume, high preoperative general memory, absence of postoperative seizures, and fewer treatment-related symptoms (Starnoni et al., 2018; Ng et al., 2019; Yoshida et al., 2020). Very few studies investigate return to work in meningioma patients, and most of them deal with this issue as a secondary outcome, reporting only the likelihood to resume a previous job (Akagami et al., 2002; Krupp et al., 2009). To our knowledge, only one study exists that specifically focuses on return to work following meningioma surgery. This article reports high tumor grade, previous history of depression, amount of sick leave in the year preceding surgery, and surgically acquired neurological deficits as significant negative predictors 2 years from surgery (Thurin et al., 2019).

Among the effects of the disease and treatment, there are indications that neurocognitive deficits contribute to the work limitations experienced by brain cancer survivors (Feuerstein et al., 2007). These problems can, in fact, influence performance in many tasks involved in work, especially for jobs requiring cognitive abilities (e.g., problem solving).

Despite the paucity of studies, going back to work is an important issue for its impact on patients' financial conditions and quality of life: Positive associations between employment and quality of life were found for patients with acquired brain injury and for cancer survivors (Main et al., 2005; Rasmussen and Elverdam, 2008; Ra and Kim, 2015; Matérne et al., 2018). Thus, return to work can be used as a surgical outcome measure. However, the research on return to work and its associated factors after brain tumor surgery is in its infancy. This study aimed to contribute to this issue through an explorative analysis of the differences between people who returned to work and those who did not after surgery, using data collected in the framework of a wider project on outcome predictors in patients with meningioma and glioma. Our hypothesis was that return to work requires a preservation of neurological, cognitive, and behavioral functions after surgery, and so the primary aim was to investigate which of these variables are associated to the resumption of work. We also investigated the difference in preoperative neurological, cognitive, and behavioral functions between patients returning to work and those who did not in order to explore if an impairment in these domains before surgery could be a potential indicator of a difficulty in postoperative returning to work. Finally, we also think that work-related variables could influence return to work, and consequently, the secondary aim of this study was to analyze the impact of professional category, working time, and home-to-work travel time on going back to work.

## Methods

### Patient Selection

We performed a retrospective study on working people from a sample of patients who were evaluated with patient-reported outcome measures (PROMs) and cognitive tests in a framework of a wider project on outcome predictors in brain tumor surgery. For this project, data were collected on patients with meningioma and glioma because they are the most frequent pathologies.

Patients who underwent tumor resection for glioma or meningioma were consecutively enrolled and participated on a voluntary basis. This project was approved by the ethical committee, and written informed consent was obtained from all patients prior to inclusion.

For the aim of this study, we selected working-age patients (18–67 years), who were self-employed or employed, undergoing tumor resection between February 2018 and June 2019. We excluded people with recurrent tumors and those undergoing a surgical procedure other than craniotomy (i.e., biopsy).

### Patient Assessment

Patients included in this study were cognitively evaluated and completed PROMs the day before surgery (T0) and after a mean time of 3 months (T1). At T0, PROMs were fulfilled at hospital admission, and at T1, they were sent to the home or through email. Together with PROMs, patients also completed a questionnaire on socio-demographic and work-related information. The cognitive evaluations were performed by a neuropsychologist at hospital admission (T0) and during the first follow-up visit (T1).

PROMs were used for the evaluation of emotional distress and disability level.

Emotional distress was evaluated with the Hospital Anxiety and Depression Scale. It is a scale for the screening of anxiety and depression states composed of 14 items: 7 for anxiety symptoms and 7 for depressive symptoms. A total score higher than 10 identifies cases with significant clinical psychological disturbances and can be used as a valid measure of emotional distress (Costantini et al., 1999).

Disability level was measured with the 12-item World Health Organization (WHO) Disability Assessment Schedule (WHODAS-12). It is composed of 12 items evaluating patients' difficulties in performing different activities due to health conditions. Each item is rated on a 1–5 scale (no difficulty, completely difficult), and the total score ranges from 0 to 100: Higher scores are indicative of higher disability levels (Andrews et al., 2009; Ustun et al., 2010).

An *ad hoc* questionnaire was built to collect socio-demographic and work-related information.

Socio-demographic data collected at T0 included age, sex, years of education, and marital status (unmarried, married/cohabitant, divorced/separated, widowed).

Work-related variables collected at T0 included working time (full time: ≥35 h/week; part time: <35 h/week), home-to-work travel time (minutes), socio-professional categories identified on the basis of the Italian classification of occupation (CP2011) (ISTAT, 2011) and grouped into blue collar (manual labor) and white collar (professional) jobs. Work-related variables collected at T1 were the following: return to previous work (yes/no), change of working time between T0 and T1 (yes/no), and change in working tasks between T0 and T1 (yes/no) for people returning to work or reasons for not going back to work (I am on sick leave/I decided to quit my job) and presence of economic difficulties due to not returning to work (yes/no).

The cognitive assessment was performed using the Montreal Cognitive Assessment test (MOCA) for the evaluation of general cognitive status and a battery of standardized neuropsychological tests: the Trail-Making Test (TMT parts A and B) for attention and executive functions (Giovagnoli et al., 1996); the 15-word Rey–Osterrieth Word List, immediate recall (ROWL-IR) and delayed recall (ROWL-DR) for memory (Caltagirone et al., 1995); and the F-A-S test for phonemic fluency (Novelli et al., 1986). The MOCA test has a total score ranging between 0 and 30: According to the normative data of a recent Italian study, scores ≤ 15.5 are considered abnormal, >17.54 indicate a normal performance, and between 15.5 and 17.54 are indicative of a borderline performance (Santangelo et al., 2015). It covers 6 cognitive domains, including visuospatial abilities, executive functions, attention, language, orientation, and memory.

Finally, the following clinical data were recorded for each patient: tumor side and location, WHO tumor grade, presence of neurosurgical complications, postoperative treatments, and functional status at T0 and T1 evaluated using the Karnosfky Performance Status (KPS) (Karnofsky and Burchenal, 1949).

### Statistical Analyses

Descriptive statistics are used to report sample characteristics, test and questionnaire scores (number and percentage for categorical variables and mean ± standard deviation for quantitative variables).

Clinical data, work-related variables, PROMs, and cognitive test scores at T0 and T1 were compared between patients who returned to work and those who did not after surgery. We decided to analyze meningioma and glioma patients separately due to the clinical and psychological differences that exist between these two pathologies. The following statistical analyses were performed: chi-squared or Fisher's exact test for categorical variables and *t*-test or Mann–Whitney *U* test for quantitative variables depending on the data distribution. The hypothesis of the normal distribution was tested using skewness and kurtosis. The level of significance of the two-tailed statistical test was set to 5% (*p* < 0.05) because this is an exploratory study and multiplicity correction is not expected (Ranstam, 2016).

Statistical analyses were conducted using SPSS 24.0 statistical software, and cases with missing data were excluded from the analyses.

## Results

**Figure 1:**
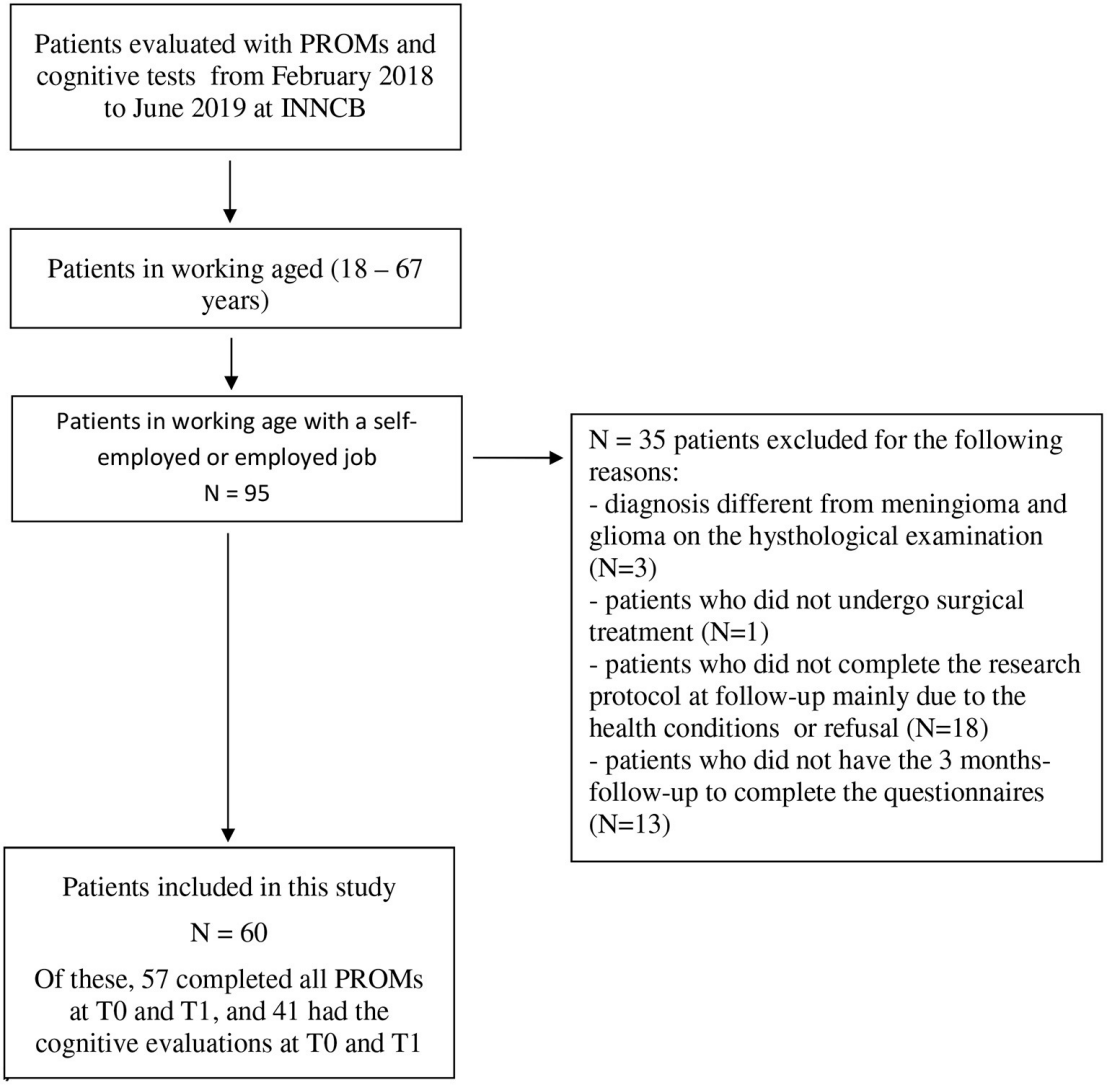
Patient selection.

A total of 60 patients were included in our study: 31 with meningioma and 29 with glioma. All patients underwent brain tumor surgery under general anesthesia except for one awake surgery. Figure 1 shows the selection of patients in working age who were self-employed or employed from the database of people evaluated with PROMs and cognitive tests. Socio-demographic, clinical, and work-related variables are reported in Table 1.

**Table 1:** Socio-demographical, clinical, and work-related variables in glioma and meningioma (number and percentage for categorical variables and mean ± standard deviation for quantitative).

**Variables T0**	**Glioma**	**Meningioma**
	**(*N* = 29)**	**(*N* = 31)**
Age (mean ± SD)	46.0 ± 11.8	48.1 ± 8.0
Gender (female)	9 (31.0%)	24 (77.4%)
Years of education	14.1 ± 3.9	15.6 ± 4.1
**Marital status**		
Unmarried	7 (24.1%)	4 (12.9%)
Married/Cohabitant	21 (72.4%)	24 (77.4%)
Divorced/Separated	1 (3.4%)	3 (9.7%)
**Tumor side**		
Right	13 (44.8%)	15 (48.4%)
Left	16 (55.2%)	15 (48.4%)
Median line	0 (0.0%)	1 (3.2%)
**Tumor location**		
Frontal	9 (31.0%)	6 (19.4%)
Parietal	2 (6.9%)	4 (12.9%)
Temporal	9 (31.0%)	2 (6.5%)
Parieto occipital	2 (6.9%)	2 (6.5%)
Fronto parietal	0 (0.0%)	6 (29.4%)
Temporo parietal	3 (10.3%)	0 (0.0%)
Insular	4 (13.8%)	0 (0.0%)
Skull base	0 (0.0%)	10 (32.3%)
Ventricular	0 (0.0%)	1 (3.2%)
**Variables T1**		
**Tumor WHO grade**		
Grade I	1 (3.4%)	22 (71.0%)
Grade II	6 (20.7%)	9 (29.0%)
Grade III	10 (34.5%)	0 (0.0%)
Grade IV	12 (41.4%)	0 (0.0%)
**Complications (Landriel-Ibanez classification)**		
Grade I (not requiring invasive treatment)	10 (34.5%)	11 (35.5%)
Grade II (requiring invasive interventions)	1 (3.5%)	1 (3.2%)
Grade III (life-threatening interventions)	1 (3.5%)	0 (0.0%)
Grade IV (death)	\	\
**Postoperative treatments**		
Radiotherapy	20 (69.0%)	0 (0.0%)
Chemotherapy	20 (69.0%)	0 0.0%)
Drug therapy	10 (34.5%)	5 (16.1%)
Rehabilitation	5 (17.2%)	4 (12.9%)
**People returning to work**	9 (31.0%)	19 (61.3%)
Change of working tasks	5 (55.6%)	3 (15.8%)
Working time reduction	4 (44.4%)	5 (26.3%)
**People not returning to work**	20 (69.0%)	12 (38.7%)
On sick leave	17 (85.0%)	9 (75.0 %)
People who quit the job	2 (10.0%)	0 (0.0%)
For other reasons	0 (0.0%)	1 (8.3%)
With economic difficulties	5 (25.0%)	0 (0.0%)

*WHO, World Health Organization; SD, standard deviation*.

In the meningioma group, 19/31 (61.3%) patients returned to work after a mean time of 3 months from surgery. Among them, 3 (15.8%) patients changed their working tasks, and 5 (26.3%) had a working time reduction. A total of 9/12 patients who did not return to work answered that they were on sick leave, and none reported economic difficulties due to not going back to work. Differences between people who returned to work and those who did not are reported in Table 2: Patients with meningioma returning to work had higher scores in KPS (*p* = 0.018), MOCA total score and language part at T1 (*p* = 0.017; *p* = 0.007), and lower scores in TMT-A and TMT-B at T0 (*p* = 0.013; *p* = 0.007) as well as WHODAS-12 (*p* = 0.019), TMT-A (*p* = 0.011), TMT-B (*p* = 0.012), and ROWL-IR (*p* = 0.021) at T1. Furthermore, most of them did not undergo postoperative treatments compared with those not returning to work (*p* = 0.002) and reported a lesser home-to-work travel time (*p* = 0.017).

**Table 2:** Comparison between patients with meningioma who returned to work and those who did not (number and percentage for categorical variables and mean ± standard deviation for quantitative).

**Clinical variables**	**Return to work**	**Non return to work**	***p*-value**
Complications			1.000
Yes	7 (58.3%)	5 (41.7%)	
No	12 (63.2%)	7 (36.8%)	
Postoperative treatments			0.002
Yes	1 (12.5%)	7 (87.5%)	
No	18 (78.3%)	5 (21.7%)	
KPS at T0	93.2 ± 10.0	91.7 ± 7.2	0.331
KPS at T1	97.2 ± 5.8	89.2 ± 13.8	0.018
**PROMs**			
HADS (Emotional distress) at T0	12.8 ± 8.0	14.1 ± 7.8	0.702
HADS (Emotional distress) at T1	10.2 ± 7.7	10.5 ± 7.6	0.865
WHODAS-12 (disability) at T0	19.0 ± 16.9	20.6 ± 17.7	0.822
WHODAS-12 (disability) at T1	12.6 ± 12.5	29.0 ± 21.9	0.019
**Cognitive tests**			
TMT–A at T0	27.7 ± 8.8	42.3 ± 13.6	0.013
TMT–A at T1	25.0 ± 6.5	42.5 ± 11.7	0.011
TMT–B at T0	82.5 ± 30.9	126.9 ± 35.6	0.007
TMT–B at T1	63.9 ± 21.7	108.5 ± 27.6	0.012
ROWL-IR at T0	40.5 ± 12.7	45.7 ± 6.8	0.351
ROWL-DR at T0	7.6 ± 4.0	8.6 ± 2.6	0.705
ROWL-IR at T1	37.4 ± 9.4	51.3 ± 6.7	0.021
ROWL-DR at T1	6.6 ± 2.9	9.9 ± 2.7	0.057
F-A-S test at T0	35.5 ± 11.0	33.9 ± 11.0	0.683
F-A-S test at T1	41.6 ± 8.9	37.3 ± 8.6	0.394
MOCA test at T0	23.1 ± 1.9	23.6 ± 1.6	0.435
MOCA test at T1	25.5 ± 1.7	23.6 ± 1.2	0.017
MOCA test: language domain at T0	5.065 ± 0.774	5.176 ± 0.466	0.859
MOCA test: language domain at T1	5.489 ± 0.386	4.862 ± 0.539	0.007
**Work-related variables**			
Socio-professional categories			1.000
Blue collar (manual labor)	2 (66.7%)	1 (33.3%)	
White collar (professional jobs)	16 (61.5)	10 (38.5%)	
Working time			0.677
Full time	13 (68.4%)	6 (31.6%)	
Part time	5 (55.6%)	4 (44.4%)	
Home-to-work travel time (minutes)	14.6 ± 12.3	33 ± 25.6	0.017

*HADS, Hospital Anxiety and Depression Scale; WHODAS-12, 12 item World Health Organization Disability Assessment Schedule; TMT, Trail Making Test; ROWL-IR, 15-word Rey–Osterrieth Word List immediate recall; ROWL-DR, 15-word Rey–Osterrieth Word List delayed recall; MOCA, Montreal Cognitive Assessment test; KPS, Karnofsky Performance Status*.

In the glioma group, 9/29 (31.0%) patients returned to work, and most of them changed their working tasks and reduced the working time (55.6%; 44.4%). A total of 17/20 patients did not return to work mainly because they were on sick leave (85.0%), and 5 (25.0%) patients reported economic difficulties because of this. Differences between people who returned to work and those did not are reported in Table 3: Patients with glioma returning to work reported lower scores in WHODAS-12 scores at T1 (*p* = 0.002) and a lesser home-to-work travel time (*p* = 0.048).

**Table 3:** Comparison between patients with glioma who returned to work and those who did not (number and percentage for categorical variables and mean ± standard deviation for quantitative).

**Clinical variables**	**Return to work**	**Non return to work**	***p*-value**
Complications			1.000
Yes	4 (33.3%)	8 (66.7%)	
No	5 (29.4%)	12 (70.6%)	
Postoperative treatments			0.310
Yes	8 (28.6%)	20 (71.4%)	
No	1 (100%)	0 (0.0%)	
KPS at T0	93.3 ± 5.0	91.0 ± 6.4	0.366
KPS at T1	92.5 ± 5.0	89.4 ± 9.4	0.588
**PROMs**			
HADS (Emotional distress) at T0	9.6 ± 7.0	12.8 ± 8.4	0.430
HADS (Emotional distress) at T1	7.2 ± 5.8	13.6 ± 10.7	0.109
WHODAS-12 (disability) at T0	7.6 ± 6.0	18.2 ± 17.4	0.076
WHODAS-12 (disability) at T1	7.4 ± 8.4	27.3 ± 18.2	0.002
**Cognitive tests**			
TMT–A at T0	36.2 ± 16.5	44.3 ± 34.2	0.906
TMT–A at T1	35.2 ± 16.7	42.3 ± 15.9	0.711
TMT–B at T0	107.3 ± 45.0	112.7 ± 71.6	0.623
TMT–B at T1	113.2 ± 65.9	147.4 ± 87.9	0.517
ROWL-IR at T0	38.7 ± 9.2	40.9 ± 10.2	0.426
ROWL-DR at T0	8.2 ± 3.6	8.1 ± 3.5	0.811
ROWL-IR at T1	41.7 ± 5.3	34.3 ± 14.1	0.365
ROWL-DR at T1	9.8 ± 0.7	6.7 ± 5.0	0.497
F-A-S test at T0	30.3 ± 11.5	38.2 ± 16.8	0.150
F-A-S test at T1	28.4 ± 9.3	32.7 ± 15.8	0.458
MOCA test at T0	23.5 ± 4.0	22.0 ± 4.5	0.450
MOCA test at T1	23.8 ± 2.7	21.0 ± 4.5	0.358
MOCA test: language domain at T0	4.876 ± 1.183	4.798 ± 0.992	0.855
MOCA test: language domain at T1	4.580 ± 1.311	4.571 ± 1.151	0.630
**Work-related variables**			
Socio-professional categories			0.546
Blue collar (manual labor)	0 (0.0%)	3 (100%)	
White collar (professional jobs)	6 (28.6%)	15 (71.4%)	
Working time			1.000
Full time	5 (25.0%)	15 (75.0%)	
Part time	1 (33.3%)	2 (66.7%)	
Home-to-work travel time (minutes)	12.1 ± 14.7	20.8 ± 14.8	0.048

*HADS, Hospital Anxiety and Depression Scale; WHODAS-12, 12 item World Health Organization Disability Assessment Schedule; TMT, Trail Making Test; ROWL-IR, 15-word Rey–Osterrieth Word List immediate recall; ROWL-DR, 15-word Rey–Osterrieth Word List delayed recall; MOCA, Montreal Cognitive Assessment test; KPS, Karnofsky Performance Status*.

## Discussion

Our study reports the rate of return to work and its associated factors after a mean time of 3 months from brain tumor surgery. We analyzed meningioma and glioma groups separately due the clinical and psychological differences between these two pathologies, including the different frequency of adjuvant postoperative treatments.

A total of 61.3% of patients with meningioma resumed their previous job. This result is similar to those of other studies in which the percentage varies from 43 to 86% although it is calculated after a longer period from surgery (Kalkanis et al., 2000; Akagami et al., 2002; Krupp et al., 2009; Schepers et al., 2018; Thurin et al., 2019). In the glioma group, 31% of patients returned to work, and this percentage is lower than in studies on low-grade glioma or mixed diagnosis (Mandonnet et al., 2015; Muto et al., 2018; Ng et al., 2019; Senft et al., 2020; Yoshida et al., 2020) but very similar to other studies on patients with glioblastoma (Gzell et al., 2014; Starnoni et al., 2018), and this result could be explained by the higher rate of high-grade glioma in our sample (75.9%). Among people returning to work, most patients with glioma and fewer than half of patients with meningioma changed their working tasks and reduced working time. Similarly, Starnoni et al. find that 61.9% of patients with glioma returned to work on a part time basis (Starnoni et al., 2018). Most people who did not return to work declared they were still on sick leave, and economic difficulties due to not working were reported only by patients with glioma. We can suppose that postoperative treatments, more frequently indicated for gliomas than meningiomas, involved costs that became more influent in a period in which the earning was lower or absent (e.g., costs for travel and accommodation for patients and their caregiver living far from the hospital; domestic help for those with disability). Moreover, patients with high-grade glioma frequently chose for themselves to quit their job due to a poor prognosis with a consequent greater financial impact.

Regarding factors associated with return to work, we found different results in meningioma and glioma groups.

Patients with meningioma returning to work were less likely to undergo adjuvant treatments and had better postoperative language, functional, and cognitive status except for immediate recall, which seems to be worse. The few studies in the literature investigating factors related to return to work in meningioma patients also report that postoperative dependency, deterioration of somatic and cognitive performance, and new neurological deficits after surgery impact the ability to resume a previous job (Kalkanis et al., 2000; Krupp et al., 2009; Thurin et al., 2019). Among preoperative variables, better attention and executive functions, evaluated with the TMT test, were found to be associated with return to work after surgery. This result should be further investigated in studies on preoperative predictors of return to work because the knowledge of these factors allows the improvement of informed consent and better responses to patients' questions about the postoperative period. Thus, if the predictive value of the TMT is confirmed in future studies, this cognitive test could be added to the other clinical scales commonly used in the preoperative assessment. In a previous study by Lee et al., the TMT-B is the only cognitive test that was predictive of 6 month progression-free survival in a sample of patients with glioblastoma (Lee et al., 2015). Finally, a novel factor that we find to be associated with return to work is the home-to-work travel time, which was less in people resuming their previous employment. This means that the distance between home and work could influence—likely together with other factors—the choice of going back to work or not. To our knowledge, home-to-work transportation is not usually studied as a factor potentially related to return to work although it could become a problem for people with new postoperative neurological deficits, such as epilepsy, motor symptoms, and fatigue and for patients without a partner, especially in the case of possible epileptic insults.

Patients with glioma returning to work reported a lower postoperative level of disability, and no significant difference was found in preoperative and postoperative cognitive functions as compared with those not returning to work. As in the meningioma group, a lesser home-to-work travel time was significantly associated with return to work in patients with glioma. However, the difference in home-to-work travel time between patients returning to work and those who did not is low, and the significance level is very close to the threshold, so this result should be interpreted with caution.

Most of the studies in the literature did not use PROMs, but investigated only socio-demographic and clinical variables as potentially associated with employment status after surgery. Similarly to us, Senft et al. do not find any association between preoperative KPS score and return to work or between postoperative treatments and return to work in patients with glioma (Senft et al., 2020). Differently from our study, Starnoni et al. report a lower preoperative KPS scores in patients with glioblastoma not returning to work at a follow-up of 6 months (78.5 vs. 85.1) (Starnoni et al., 2018); Muto et al. find a lower median postoperative KPS in patients with low-grade glioma unable to work at a follow-up of 6.9 months (90 vs. 100) (Muto et al., 2018); and Yoshida et al. report high preoperative general memory scores among the predictive factors of the return to work at 1 year after surgery (Yoshida et al., 2020). In our study, the professional category was not associated with return to work, but conflicting results exist in the literature on the influence of these variables (Starnoni et al., 2018; Ng et al., 2019; Senft et al., 2020; Yoshida et al., 2020). Emotional distress was also not significantly associated with going back to work even if we can observe worse scores in patients with glioma who did not return to work.

The different results found in patients with meningioma and glioma could be related to the specific characteristics of these two diagnoses. All patients with glioma underwent postoperative treatments, and these could have prevented return to work within 3 months regardless of other variables, e.g., cognitive performance that was not so different in those who returned to work and those who did not compared with the meningioma group in which cognitive scores seem to be a discriminative variable even if most of the patients had cognitive scores in the normal range. In particular, the TMT test seems to be the most sensitive, probably because it requests multiple cognitive processes involving mental flexibility and multitasking skills. The 3 month follow-up is probably too early for patients with glioma who tend to return to work after a longer period of time (Senft et al., 2020). Moreover, the group of patients with glioma is composed of both grade I/II and grade III/IV glioma that have different prognoses, and consequently, future studies on larger samples should consider these pathologies separately. Future research is also needed to explore if a more complex symptom network and an interaction between specific clinical, cognitive, and psychological variables can influence the return to work rather than single factors.

Our study has some limitations that prevent us from reaching a strong conclusion on the factors associated with return to work after brain tumor surgery: small sample size, the lack of a second follow-up (e.g., at 6 months or 1 year) that could be informative in particular for patients with glioma who usually have a longer postoperative care pathway, the subjective evaluation of economic difficulties due to not returning to work, and the explorative nature of our study that allowed us to investigate only the association between patients' characteristics and postoperative employment and not the direction of this relationship and the variables' predictive value of return to work; moreover, due to the explorative nature of our study, we did not correct the *p*-value despite the multiple comparisons, and consequently, some significant results should be further investigated. Finally, other variables could play an important role in returning to work, e.g., factors related to the workplace (relationship, presence of barriers, colleagues and employers' attitudes).

The results of our study, even if preliminary, can be useful in both clinical and research contexts. The knowledge of factors influencing return to work allows identifying patients who could have difficulties at work after surgery and planning tailored interventions to support and facilitate the resumption of previous working tasks. Our study reports additional variables to the existing literature that could be taken into account in future studies that aim at investigating factors related to return to work after brain tumor surgery. Both preoperative and postoperative variables (clinical, patient-reported, cognitive, related to work) should be used in larger samples to build predictive models using return to work as a specific outcome measure of surgical treatment.

## Conclusion

A total of 61.3% of patients with meningioma and 31.0% of patients with glioma resumed their previous job after a mean time of 3 months from tumor resection. Frequently, return to work is characterized by changes in working time and tasks, in particular in patients with glioma. The factors associated with return to work in patients with meningioma were better preoperative and postoperative scores in attention and executive functions, better postoperative functional and cognitive status, lower postoperative disability level and frequency of adjuvant treatments, and lesser home-to-work travel time. Patients with glioma who returned to work had a lower postoperative level of disability and lesser home-to-work travel time.

The evaluation of factors influencing the return to work with specific questionnaires and tests allows clinicians to plan specific interventions, e.g., vocational rehabilitation programs, for people with disability and cognitive difficulties. Furthermore, the knowledge of these factors could be used during the preoperative evaluation to better respond to patients regarding their questions on return to work after surgery.

## Data Availability Statement

The raw data supporting the conclusions of this article will be made available by the authors, without undue reservation.

## Ethics Statement

The studies involving human participants were reviewed and approved by Comitato Etico Fondazione IRCCS Istituto Neurologico Carlo Besta. The patients/participants provided their written informed consent to participate in this study.

## Author Contributions

SS contributed to the acquisition, analysis, and interpretation of data and drafting of the manuscript. AM participated in acquisition of data and drafting of the manuscript. FA, MS, AF, and FD participated in the acquisition of data. ML contributed to the concept and design of the project. PF and MB contributed to the concept and design of the project and the acquisition of data. All authors contributed to the revision of the manuscript and approved the final version for submission.

## Conflict of Interest

The authors declare that the research was conducted in the absence of any commercial or financial relationships that could be construed as a potential conflict of interest.

## Abbreviations

T0: before surgery
T1: after 3 months from surgery.
